# Itrax micro X-ray fluorescence (μXRF) for soft biological tissues

**DOI:** 10.1016/j.mex.2018.10.001

**Published:** 2018-10-06

**Authors:** Patricia Gadd, Karthik Gopi, Jesmond Sammut, Neil Saintilan, Jagoda Crawford, Debashish Mazumder

**Affiliations:** a Australian Nuclear Science and Technology Organisation, Locked Bag 2001, Kirrawee DC, NSW, 2232, Australia; b Centre for Ecosystem Science, School of Biological, Earth and Environmental Sciences, The University of New South Wales, UNSW Sydney 2052, Australia; c Department of Environmental Sciences, Macquarie University, Sydney, NSW, Australia

**Keywords:** Determining the elemental profile of soft biological tissue samples through Itrax, Itrax, Biological samples, Elemental profile, Micro X-ray fluorescence

## Abstract

Determination of the elemental composition of soft biological tissue is a time-consuming and tedious process when using traditional analytical techniques. In this method, micro X-ray fluorescence (μXRF) via Itrax, a scanning instrument, was used to determine elemental abundance at a resolution of 200 μm. Itrax μXRF was initially designed for elemental profiling of geological cores, and the capability of this technique was extended to soft biological tissue samples. The samples were dried and ground into a fine powder before analysis. The scanner generates elemental values as counts per 1 mm and these values are standardised to obtain the relative elemental abundance of the elements present in the samples. The acquired data can be used for environmental and biological research.

• No literature could be found whereby the capability of Itrax μXRF has been extended to soft biological tissue samples.
• The major advantages Itrax has over conventional methods is that it is a simultaneous technique which allows data to be acquired for over 30 elements at once with minimal sample preparation.
• It is a non-destructive process where the samples can be re-used for additional analyses if necessary; this is especially useful when there is only a limited amount of sample available for other analyses.

## Graphical abstract

**Figure:**
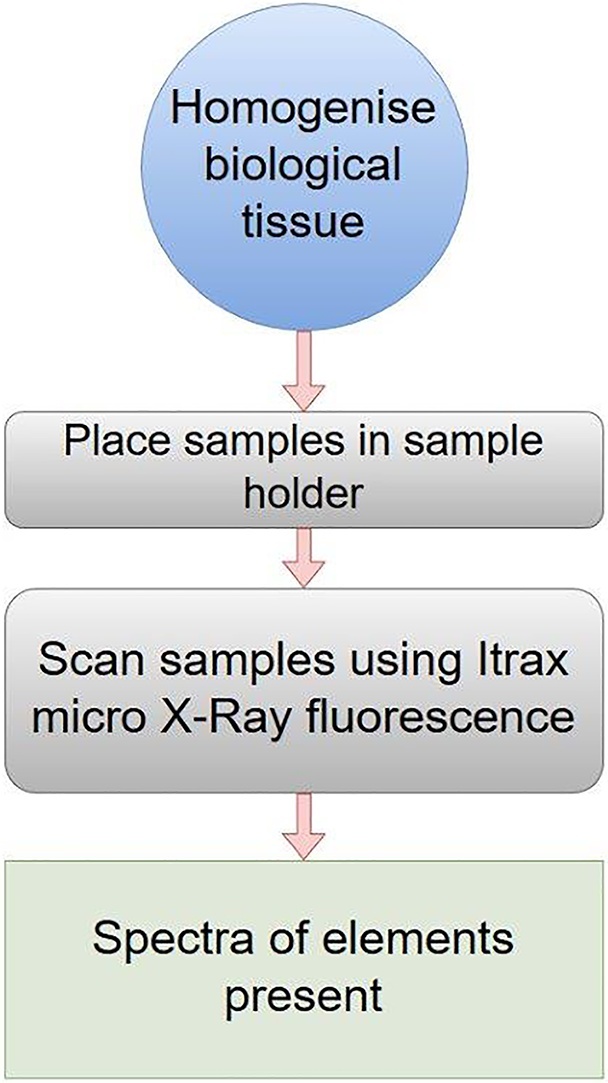

**Specifications table**

**Table:** 

**Subject Area**	*Environmental Science*
**More specific subject area:**	*Elemental profiling of soft biological tissues*
**Method name:**	Determining the elemental profile of soft biological tissue samples through Itrax.
**Name and reference of original method**	To our knowledge there are no standard methods available for scanning soft biological tissue samples through Itrax.
**Resource availability**	

## Method details

### Background

The use of elemental profiling, in conjunction with other analyses, has become prevalent in food provenance work. The elemental composition of *Pampus argenteus*, *Trichiurus lepturus*, *Pseudosciaena polyactis* and squid was used to distinguish between three different geographic origins through a combination of statistical models [1]. Similarly, Li et al. [5] found that elemental composition can be used to distinguish between different production methods, i.e. farmed vs wild-caught. These studies show that elemental profiling can play a vital role in seafood provenance. The methods used in these studies were: inductively coupled plasma mass spectrometry (ICP-MS), Atomic emission spectrometry and Inductively coupled plasma atomic emission spectroscopy (ICP-AES). These methods require the samples to be prepared using digestion before analysis. Here we describe a novel use of Itrax micro X-ray fluorescence (μXRF), a scanner capable of determining the elemental abundance of samples at a resolution of 200 μm. The data obtained from the Itrax μXRF were successfully used to distinguish the geographic origin as well as the production methods of *Penaeus monodon* from various regions in the Asia-Pacific [4]. Using Itrax μXRF has several advantages over the conventional methods; it requires minimal sample preparation and it is non-destructive allowing the sample to be used for additional analyses. While this method was originally intended for determining the elemental abundance of soil cores, using it for biological matrices has proven to be successful.

#### Sampling

Tiger prawn (*Penaeus monodon*) samples used in this application were collected from the Sydney Fish Market, NSW, Australia. Samples were placed in resealable plastic bags, labelled and stored in a cooler before being transported to the Australian Nuclear Science and Technology Organisation (ANSTO) laboratory. The samples were frozen until the analysis took place.

#### Sample preparation

The samples were removed from the freezer and thawed. The exoskeleton of the prawns was removed along with the hindgut and gonads, and then rinsed with deionized water to remove any contaminants. A 5 cm^2^ section of abdominal tissue was removed from the ventral side of the prawn with a clean stainless-steel scalpel. The tissue was rinsed with deionized water again and then transferred into a clean, dry petri dish using clean forceps. The petri dish was labelled and then placed in an oven at around 60 °C for 48 h to ensure that the samples were completely dry. The dry samples were subsequently ground into a fine powder to ensure homogeneity using a mortar and pestle [2], which was cleaned with 70% ethanol between each sample. The fine powder was then transferred into a clean glass vial and capped to prevent moisture from entering the powdered samples. The glass vials need to be stored in a desiccator to prevent moisture absorption. For instances when long-term storage of the sample is needed, they should be vacuum-packed and stored in a freezer between −17 to −20 °C.

#### Alternative sample preparation

As Itrax μXRF is based on a surface scan which outputs a semi-quantitative count of the elements present in the sample, it is possible to scan complete samples. The output from complete samples will show the places where certain elements are concentrated. For this study homogenized samples were used because the concentration of certain elements in different parts of the soft biological tissue was not as necessary for provenance; the associated research required the relative abundance of elements.

Setting up the samples for scanning through Itrax

**Fig. 1:**
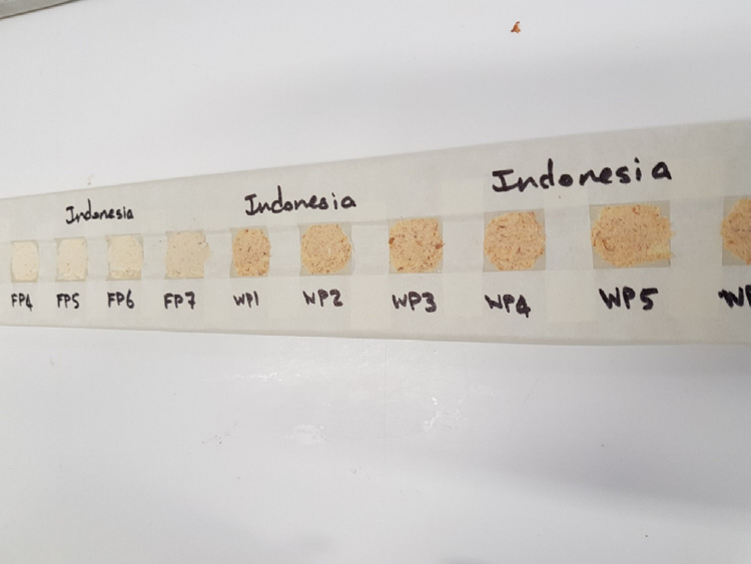
Grid samples as described in step 7 and 8 completed.
7) Transfer around 2 g of the powdered sample into a square each and label the masking tape (Fig. 2).

1) Through trial and error of different methods we determined that a Perspex sample holder, normally used for wood samples, was ideal for holding our powdered samples.
2) Clean the sample holder using ethanol.
3) Place a layer of clear double-sided sticky tape across the middle of the sample holder.
4) Measure out 2 cm intervals on the double-sided tape and place around 1 cm of masking tape in between the intervals.
5) Leave a gap of 0.5 mm from the double-sided tape and cover the top and bottom of the sample holder with masking tape, leaving a gap of around 2 cm in between.
6) Once complete, there should be a grid of squares that are roughly 4 cm^2^ (Fig. 1).

**Fig. 2:**
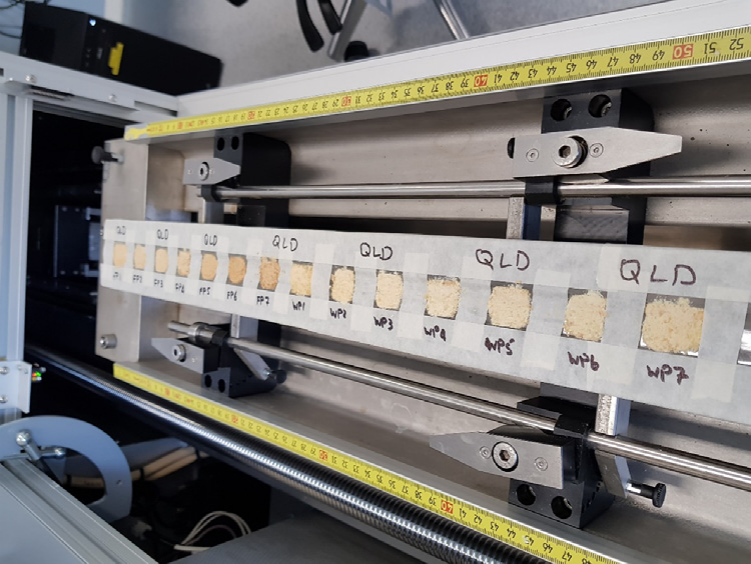
Sample holder mounted on Itrax instrument, following step 9.
8) Flatten the samples to approximately 2 mm in thickness.
9) Transfer the sample holder to the Itrax machine (Fig. 2).

#### Scanning samples using Itrax

The Molybdenum target tube worked best for these samples. The samples were continuously scanned at 1 mm intervals with 10 s exposure time. The current used was 55 mA and voltage was 30 kV. Each sample holder held 14 samples. The scans for the masking tape were clearly distinct from the samples and were removed from the dataset (in this case the tape had a lower count of magnesium than the organic samples). This saved time with scheduling the samples in the Itrax core scanner software and reduced the data processing time after the scans were completed.

#### Processing results and validation of methods

The relative abundance of elements in the samples was given as counts at 1 mm intervals. The data were standardized to give the relative abundance of every element present in the sample. All 20 data points were acquired for each sample which was averaged to minimize any effects of heterogeneity.

Neutron Activation Analysis (NAA), which produces quantified values, was used to validate the semi-quantitative trends from Itrax. To test the reproducibility of these results, around 60 individual *P. monodon* samples were scanned and the results were validated using NAA.

The standardized data can be used for a range of statistical analyses depending on the objectives of the research. For our purpose, the elemental data were used to determine the provenance of tiger prawns (*P. monodon*); see details in Gopi et al. [4].

### Quality control and assurance

Micro-XRF has bee utilised widely in environmental studies since its inception [3]. Geological studies typically use μXRF to determine the elemental composition of soils and rocks [7]. Ramsey et al. [6] found that using μXRF was comparable to ICP-AES for the major elements on the periodic table for silicate rocks. The consistent use of μXRF for geological studies show that the method is reliable and accurate for determining the relative elemental abundance of samples. However, for future studies we will be considering the use of a bulk batch of quality control samples to ensure accurate detections of elemental counts. A comparison between the Itrax scanner and ICP-MS should be conducted in the future to test the detection limits of μXRF for biological samples.

### Conclusion

The modified method shown here has potential as a tool for food provenance researchers and other end-users. It is an easy and rapid method which can be used to determine the relative elemental abundance of soft biological tissues. Analysing samples through Itrax μXRF allows the samples to be saved for additional analyses. This is vital when samples are limited, and additional analyses might need to be conducted, especially in terms of food quality or safety.
